# 

**Published:** 1916-02

**Authors:** 


### OUR NEW SUBSCRIBERS

| | |
|---|---|
| Dr. L. B. Alen | Ala. |
| Dr. C. S. Taylor | Ala. |
| Dr. C. B. Ingram | N. C. |
| Dr. H. R. Carson | Ariz. |
| Dr. V. D. Washburn | Del. |
| Dr. Willis R. Moss | Ky. |
| Dr. P. H. Singleton | Ky. |
| Dr. A. C. Jordon | Ark. |
| Dr. C. F. Crokrite | Texas |
| Dr. J. C. Bell | N. C. |
| Dr. C. H. Carter | Texas |
| Dr. L. C. Preson | Okla. |
| Dr. J. L. Hare | Ark. |
| Dr. Henry Leidhemier | Lr. |
| Drs. Ferguson & Foley | Ky. |
| Dr. A. A. Parker | Md. |
| Dr. J. T. Harrington | Texas |
| Dr. G. McClanahan | La. |
| Dr. J. E. Parramore | Okla. |
| Drs. Harrell & Rawls | Va. |
| Dr. T. J. Coble | Tenn. |
| Dr. N. T. McDaniels | Ga. |
| Dr. C. G. Todd | S. C. |
| Dr. J. E. Suggs | Md. |
| Dr. W. V. Batson | Okla. |
| Dr. J. A. R. Moseley | Texas |
| Dr. Jas. B. Darden | W. Va. |
| Dr. M. A. Blanton | Tenn. |
| Dr. H. T. Hopewell | Va. |
| Dr. G. E. Cannon | Ark. |
| Dr. Thos. Douglas | Ark. |
| Dr. H. W. Hatcher | Ark. |
| Dr. T. Johnson | Md. |
| Dr. W. R. Williams | Va. |
| Dr. E. T. Swann | Ala. |
| Dr. J. A. Hinuncutt, Jr. | Ga. |
| Dr. A. R. Howell | Ark. |
| Dr. H. H. Rhoads | Texas |
| Dr. W. W. Hale | Ark. |
| Dr. L. O. Vaughan | Va. |
| Drs. Campbell & Hayes | Ark. |
| Dr. C. T. Hayden | W. Va. |
| Dr. S. J. Ozment | Ark. |
| Drs. Faust & Vaden | Okla. |
| Dr. J. B. Mason | Ky. |
| Dr. E. L. Dawson | Okla. |
| Dr. C. G. Bell | Miss. |
| Dr. N. J. Latimer | Ark. |
| Dr. J. M .King | Ark. |
| Dr. L. S. McMullin | Tenn. |
| Dr. H. H. Workman | S. C. |
| Dr. V. D. Washburn | Del. |
| Dr. D. L. Jones | W. Va. |
| Dr. J. M. Doss | Texas |
| Dr. W. W. Chaffin | Va. |
| Dr. A. L. Wilson | Va. |
| Dr. W. S. Stille | W. Va. |
| Dr. W. R. Bennett | Texas |
| Dr. R. W. Williams | Okla. |
| Dr. A. E. Sweatland | Texas |
| Dr. W. A. Wallace | S. C. |
| Dr. A. L. Franklin | Ark. |
| Dr. R. C. Wolfe | Ark. |
| Dr. H. R. Drew | Fla. |

Dr. T. D. Frizzell ___________Texas

Dr. Walter Dotson _________Tenn.

Dr. C. J. Baumgartner ______Fla.

Dr. W. M. Gaillard _________S. C.

Dr. H. G. Nicholson ______W. Va.

r. S. M. Wagaman ________Md.

Drs. Elder & Nevitt ______Texas

Dr. A. J. Cox _____________Texas

Dr. R. C. Hanna _____________Ala.

Dr. J. H. Lander _________Texas

Dr. M. G. Elliott __________S. C.

Dr. G. H. Garrett ________Texas

Dr. W. T. Sappington ________Md.

Dr. T. F. Spurgeon _________Okla.

Dr. Sam Lindsay ___________S. C.

Dr. J. E. Vaughan __________Va.

Dr. V. D. Holoway ________Tenn.

Dr. I. P. Burdine __________Miss.

Dr. L. F. Bonner ___________S. C.

Dr. Jas. J. Phillips ________N. C.

Dr. A. W. Pleasants ___________Va.

Dr. J. R. Pharr __________W. Va.

Dr. Jas. T. Shuler __________Okla.

Dr. I. D. Winston ___________Ky.

Dr. W. H. Smith _____________Ky.

Dr. Z. K. Jones _______________Ky.

Dr. R. J. Gauldin __________Texas

Dr. J. R. Miller _____________S. C.

Dr. H. L. Proctor ___________Fla.

Dr. C. I. Cain _______________Ga.

Dr. J. A. Gilmer _____________Va.

Dr. J. H. Phillips __________Texas

Dr. S. O. Smith ____________Miss.

Dr. E. J. Stahl __________W. Va.

Dr. L. D. Berryman ________Ark.

Dr. J. G. Edwards ___________Okla.

Dr. T. B. Lewis _____________Miss.

Dr. M. J. Carson ___________N. C.

Dr. J. A. Hatchett ________Okla.

Dr. J. F. Covington ___________Ga.

Drs. Williams & Edwards__Texas

Dr. H. O. Huffman __________Okla

Dr. H. O. Wyneken ________Texas

Dr. T. S. Barkley __________Texas

Dr. B. H. Gilmer __________N. C.

